# Supplementary material for: Relative Strength Variability Measures for Brain Structural Connectomes and Their Relationship With Cognitive Functioning
Source: Hum Brain Mapp. 2025 Aug 6;46(11):e70314. doi: 10.1002/hbm.70314 (PMC12326445; doi:10.1002/hbm.70314)
Supplement: Supplementary file 1 — Data S1: Supporting Information. [file HBM-46-e70314-s001.pdf]

Relative strength variability measures for brain structural connectomes and their relationship with cognitive functioning
Supplementary Materials

Hon Wah Yeung[1], Colin R. Buchanan[1], Ian J. Deary[1], Elliot M. Tucker-Drob[3,4], Mark E. Bastin[1,5], Heather C. Whalley[2], Keith M. Smith[6,*], and Simon R. Cox[1,*]

[1] Lothian Birth Cohorts, Department of Psychology, University of Edinburgh, Edinburgh, United Kingdom
[2] Department of Psychiatry, University of Edinburgh, Edinburgh, United Kingdom
[3]Department of Psychology, University of Texas, Austin, TX, USA
[4]Population Research Center and Center on Aging and Population Sciences, University of Texas at Austin, TX, USA
[5]Centre for Clinical Brain Science, University of Edinburgh, Edinburgh, United Kingdom
[6]Department of Computer and Information Sciences, University of Strathclyde, Glasgow, United Kingdom
[*]These authors share joint senior authorship

July 9, 2025

A hRSV as a Weighted Statistical Complexity measure

A weighted measure of statistical complexity of networks should agree with the properties detailed for the binary case in (Smith and Smith, 2024). Namely, it should be 0 for regular graphs, tend to 0 for Erdös-Rényi graphs in the thermodynamic limit (as $n \to 0$) and be lower for more randomised models such as random geometric graphs and configuration models of networks. Here, we demonstrate that these properties are not all satisfied for RSV, but are all satisfied for hRSV with a $2\%n$ sliding window.

Firstly, regularity in a weighted network would entail the network had weights which were all equal. In this case, the relative strength of all nodes would be 1, for node i, the denominator being the mean of neighbourhood node strengths would be equal to the node strength of node i, so all relative node strengths would be equal and their variance would therefore be 0.

Now, we assess the behaviour of the metrics with respect to different weighted random models– Erdös-Rényi (ER) random graphs with weights $w_{ij} \sim U[0,1]$, random geometric graphs where weights are inverse distances $w_{ij} = exp(-d(i,j))$ for $d(i,j)$ the Euclidean distance between nodes i and j, and random heterogeneous geometric graphs (Smith et al., 2019), where weights are $w_{ij} = exp(-d(i,j))(x_i + x_j)$ with $x_i \sim LN(\mu, \sigma)$, such that the shape parameter σ controls the degree heterogeneity in the network. For comparisons with other models we use the default parameters $\mu = 0.5$, $\sigma = 0.2$. Finally, we also study the configuration models of the heterogeneous geometric graphs. For this, the underlying binary networks are submitted to the standard configuration model procedure (links are disconnected leaving each node of degree k_i with k_i stubs which are then randomly paired across the network to create new links) and then the k_i weights of node i of degree k_i are randomly assigned to the new randomised links of node i. The results for RSV and hRSV of these models with varying network densities ($d = 0.05, 0.1, \ldots, 0.95$), and network sizes ($n = 1000, 2000, \ldots, 10000$), averaged over 100 iterations, are reported in Figure A.1.

We see that hRSV replicates the behaviour of the binary normalised hierarchical complexity measure (Smith and Smith, 2024), with hRSV of ER random graphs tending to 0 with increasing n, and heterogeneous geometric graphs having markedly

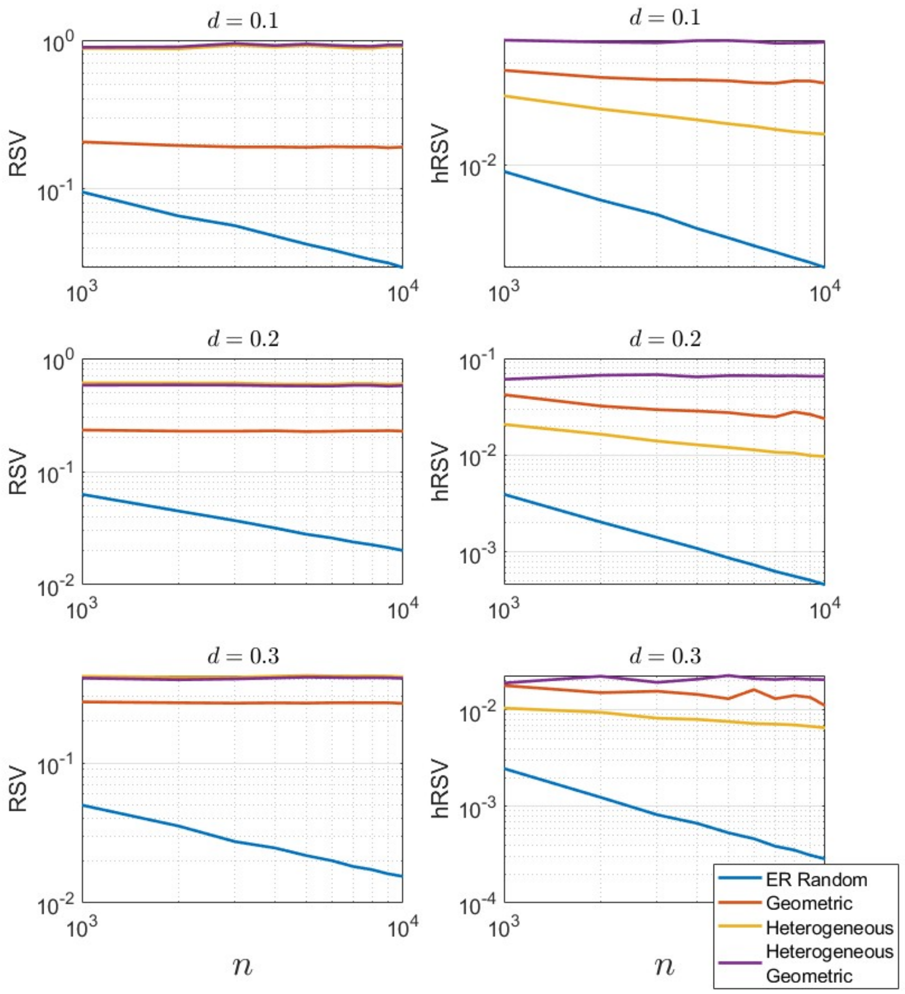

Figure A.1: The behaviour of Relative Strength Variability (RSV) and hierarchical RSV (hRSV) with respect to random weighted network models with varying size n (on the x-axis) and density d (as indicated in plot titles). Models shown are Erdös-Rényi random graphs (ER random), random geometric graphs (Geometric), random heterogeneous graphs (heterogeneous) and random heterogeneous geometric graphs (heterogeneous geometric), as in the legend. Values are averages over 100 model iterations.

larger values than their configuration models (random heterogeneous graphs) and random geometric graphs. While RSV also appears to tend to 0 for ER random graphs, it is clear that it does not distinguish well between heterogeneous geometric graphs and their configuration models, with these models having very similar values of RSV in all cases. Taken together, we can propose hRSV as a measure of statistical complexity for weighted networks, but not RSV.

Notably, both RSV and hRSV appear to be well normalised with respect to network size for heterogeneous geometric graphs. This is important because these graphs are good models of real-world networks which indicates the measures can be used straightforwardly to compare real-world networks of different sizes.

The behaviour of the metrics with respect to network degree heterogeneity was then explored. Specifically, in Figure 4 in Smith and Smith (2024), the binary measure of statistical complexity peaks with $\sigma \in [0.2, 04]$. We replicate this using hRSV in weighted networks with a suitably small window size w. Here, the parameter σ, which controls the node strength heterogeneity in the heterogeneous geometric graphs, is varied. Values are computed 100 times for each $\sigma = 0.05, 0.1, \ldots, 1$, density $d = 0.3$ (matching the structural connectomes), and sliding windows $w = 5, 10, 25, 50, 125, 250, 500$. Results are shown

in Figure A.1 for $n = 1000$ and $n = 85$. Here, we vary the window size for hRSV from $n/2$ to $\sim 2\%n$, with all choices shown in the legends.

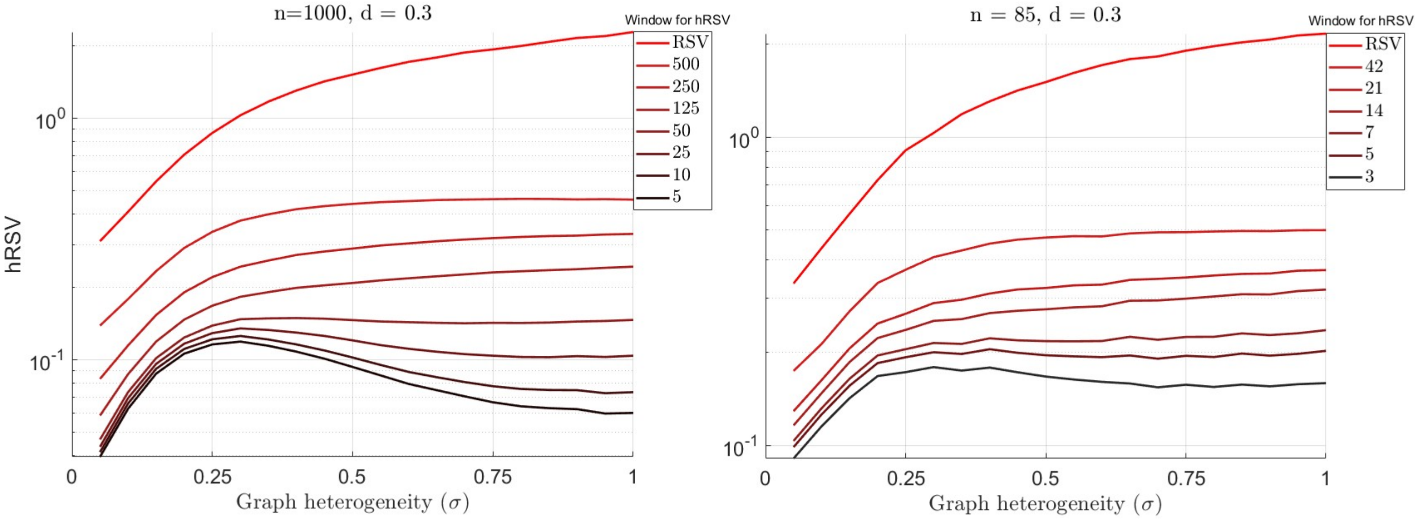

Figure A.2: The behaviour of hierarchical Relative Strength Variability (hRSV) measures with respect to geometric network degree heterogeneity.

For small w we replicate the behaviour seen for normalised hierarchical complexity in (Smith and Smith, 2024) with the highest values achieved for graphs with $\sigma \in [0.2, 0.4]$. Interestingly, for RSV the values continue to increase with increasing σ, while for intermediate w the prevailing behaviour is a levelling off with large σ. This further motivates the selection of a suitably small w for the real world applications described in the results, to best distinguish the hRSV measure from the standard RSV.

A.1 Figures showing correlations among the graph measures for 6 different network weights

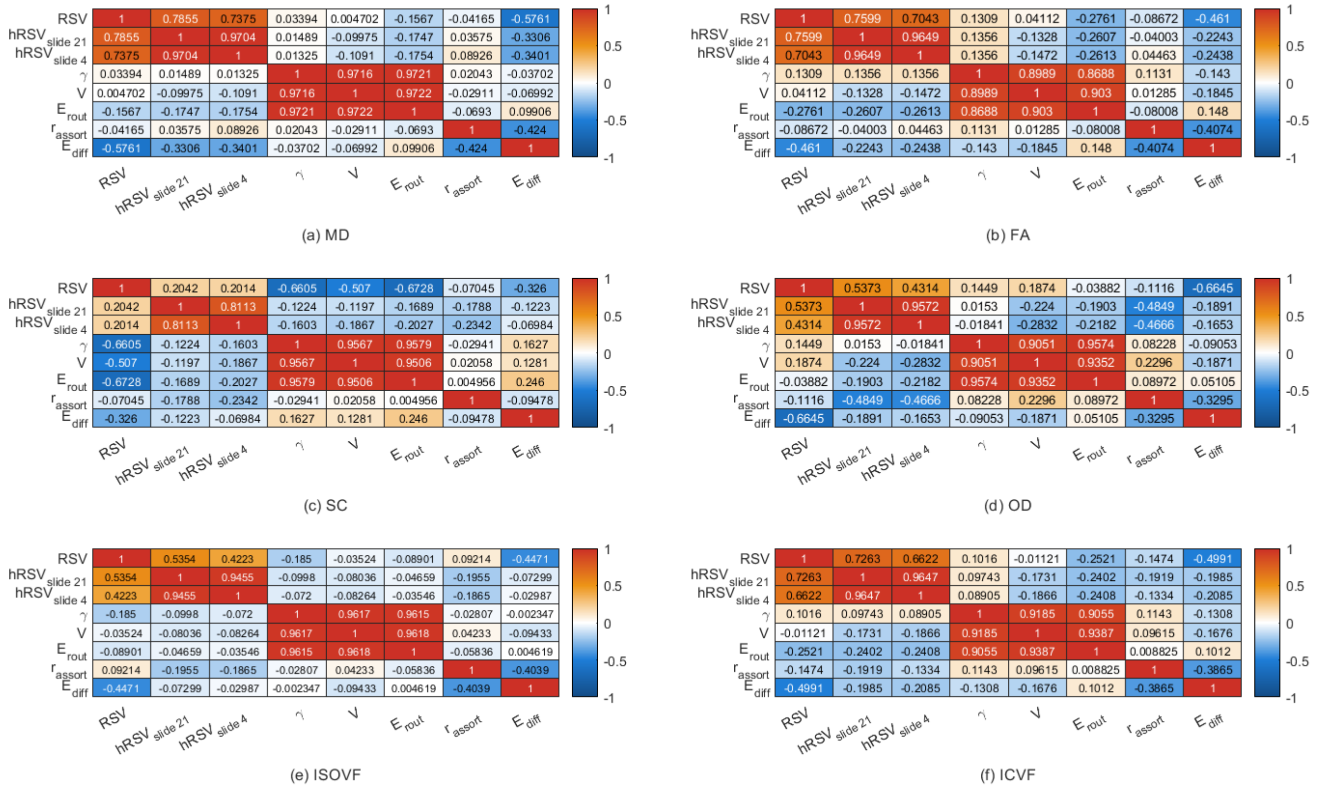

(a) MD (b) FA (c) SC (d) OD (e) ISOVF (f) ICVF

Figure A.3: Heatmaps showing correlation among the five chosen graph metrics based on different connectome weights. RSV = Relative Strength Variability, hRSV$_{slide_n}$ = Relative Strength Variability sliding window variant where n is the window size, γ = Clustering Coefficient, r_{assort} = assortativity, V = node Strength Variance, E_{diff} = Global Diffusion Efficiency, E_{rout} = Global Routing Efficiency

B Figures showing age- and sex-corrected associations between g-factor and graph measures

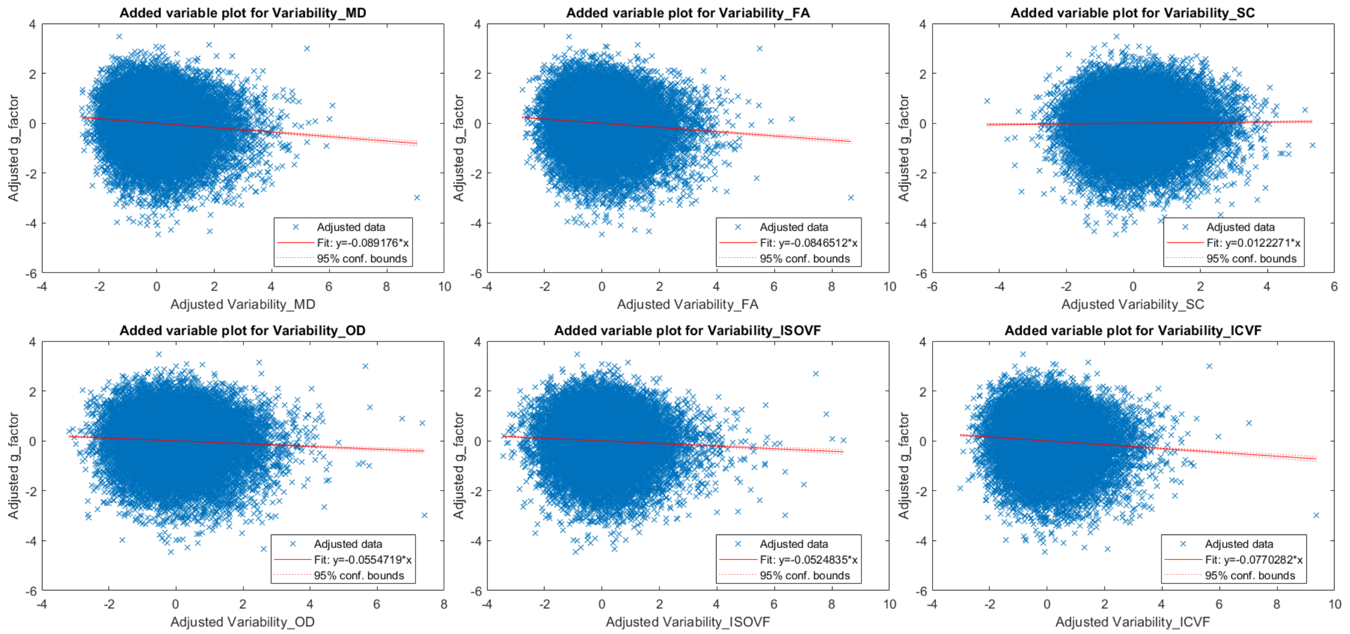

Figure B.1: Scatter plots showing the associations between the variability measure and the g-factor, adjusted for common covariates, for each of the six network weights.

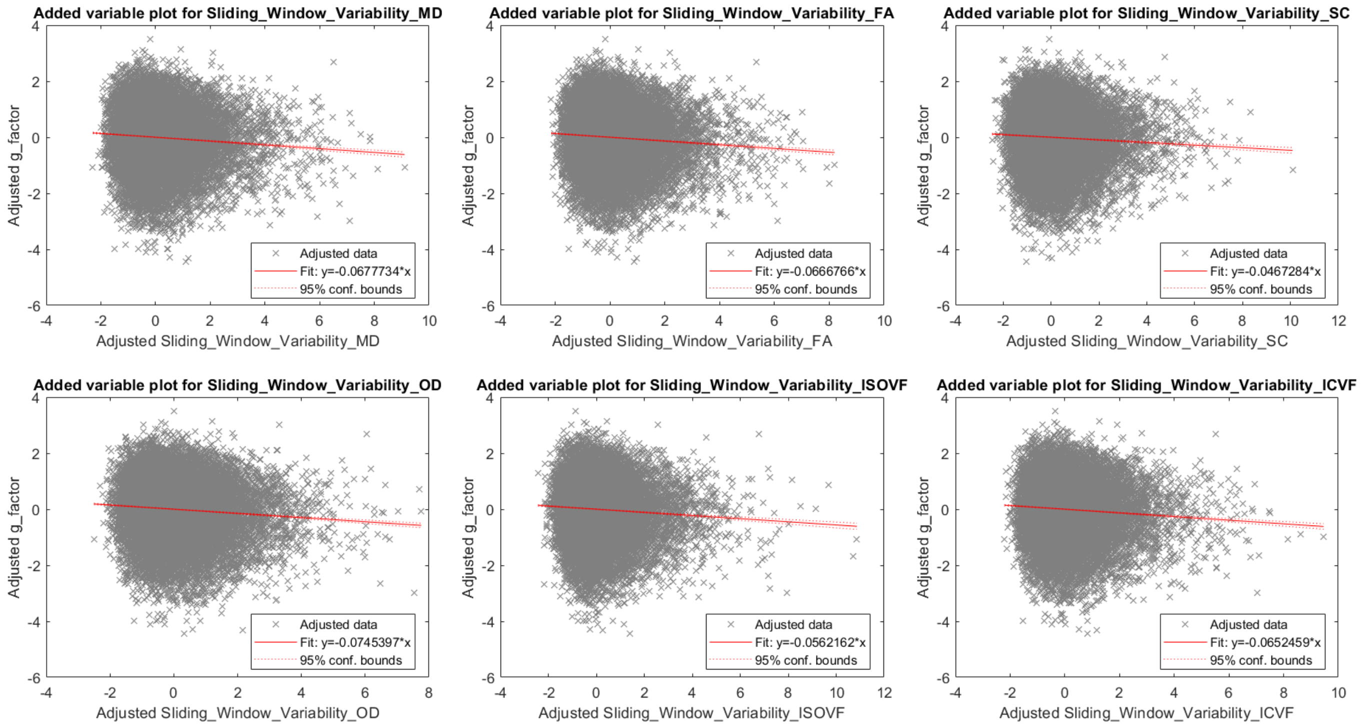

Figure B.2: Scatter plots showing the associations between the fine sliding window variability measures and the g-factor, adjusted for common covariates, for each of the six network weights.

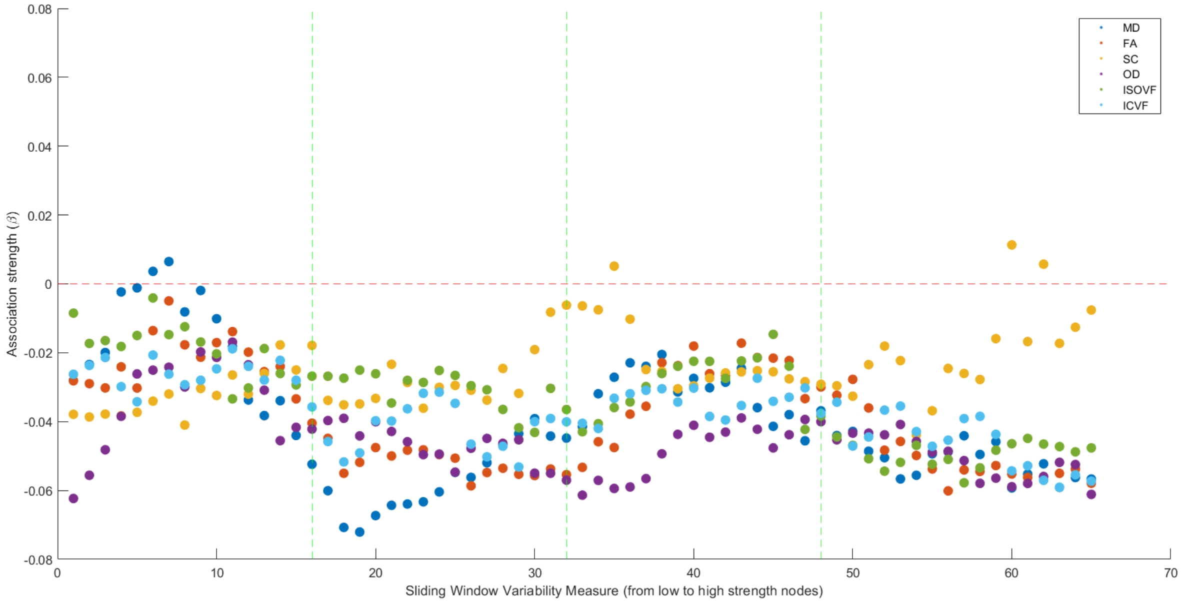

Figure B.3: Scatter plots showing the associations between the fine sliding window variability measures and the g-factor, adjusted for common covariates, for each of the six network weights.

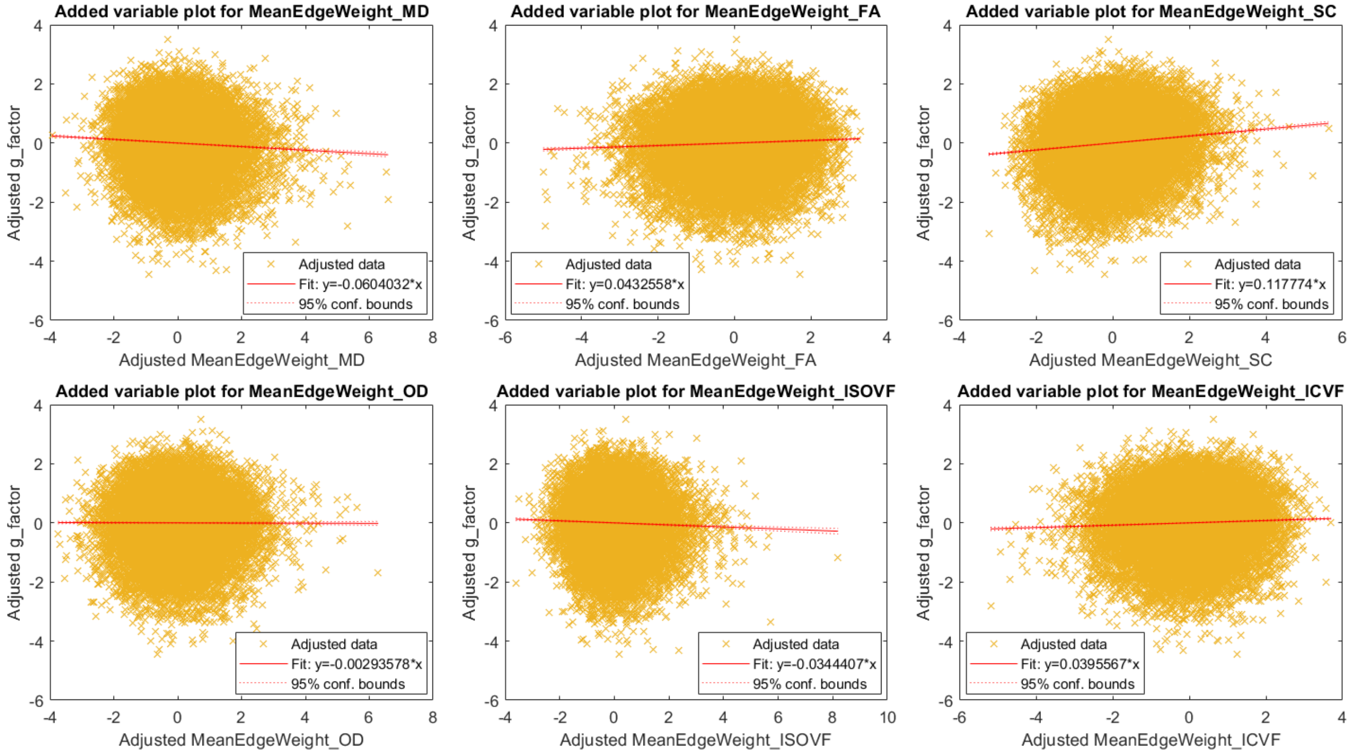

Figure B.4: Scatter plots showing the associations between mean edge weight and the g-factor, adjusted for common covariates, for each of the six network weights.

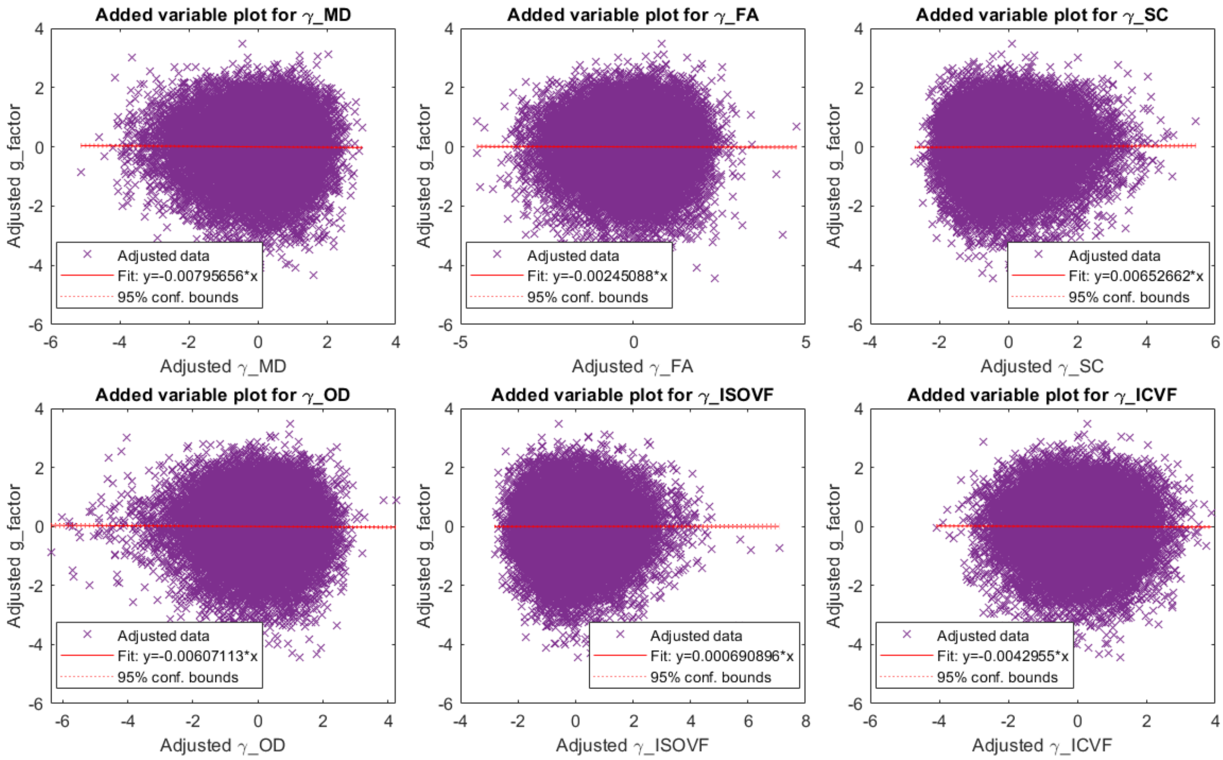

Figure B.5: Scatter plots showing the associations between clustering coefficients and the g-factor, adjusted for common covariates, for each of the six network weights.

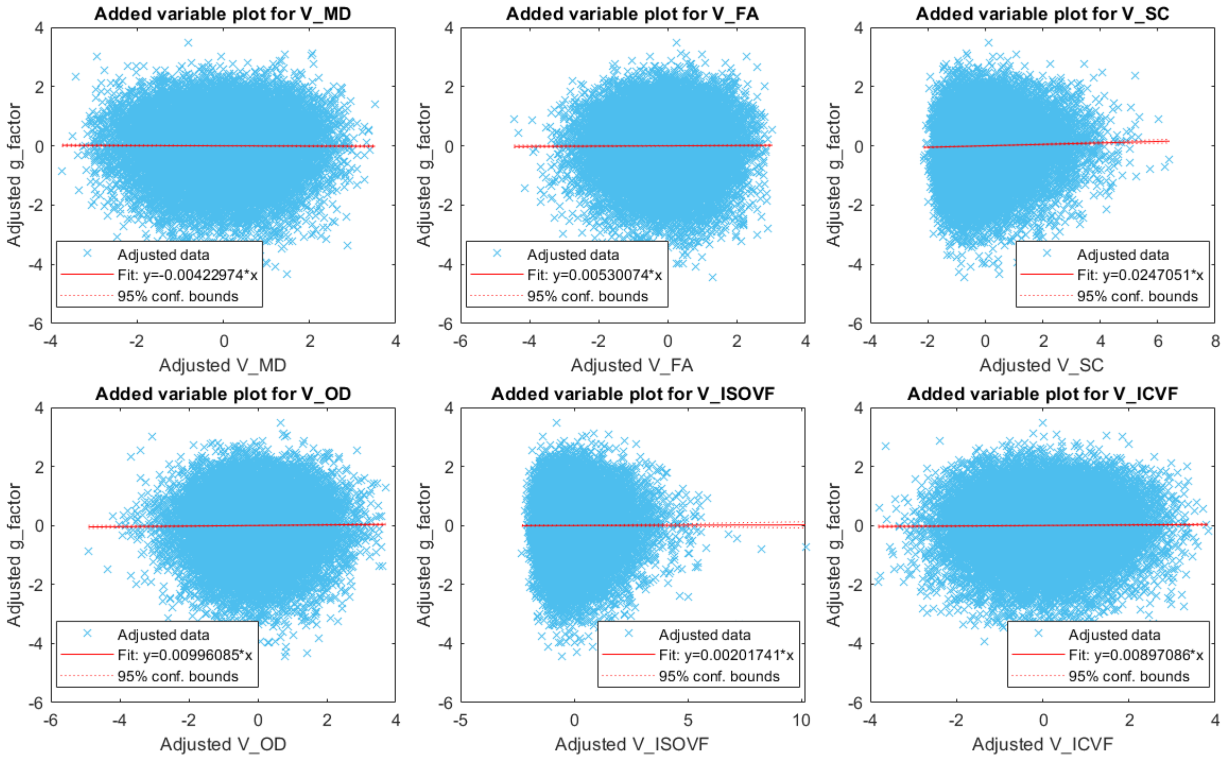

Figure B.6: Scatter plots showing the associations between strength variance and the g-factor, adjusted for common covariates, for each of the six network weights.

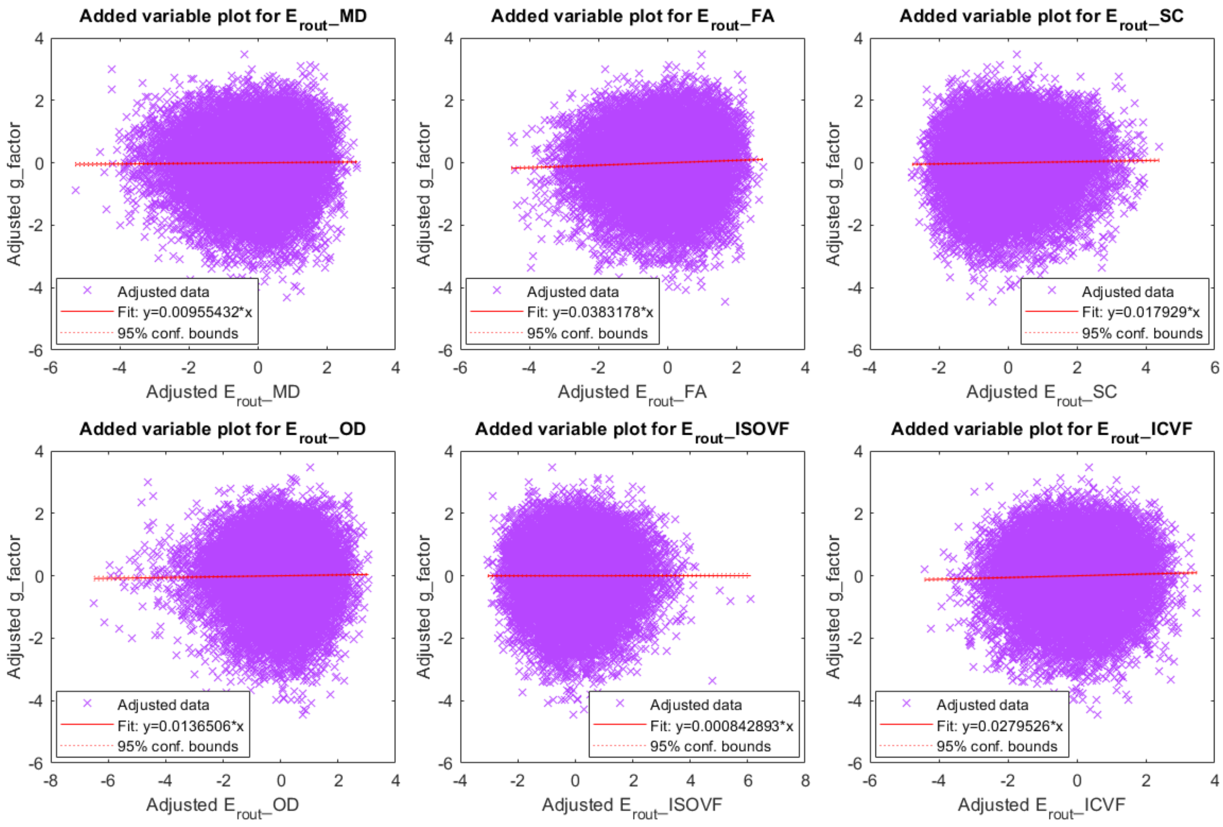

Figure B.7: Scatter plots showing the associations between routing efficiency and the g-factor, adjusted for common covariates, for each of the six network weights.

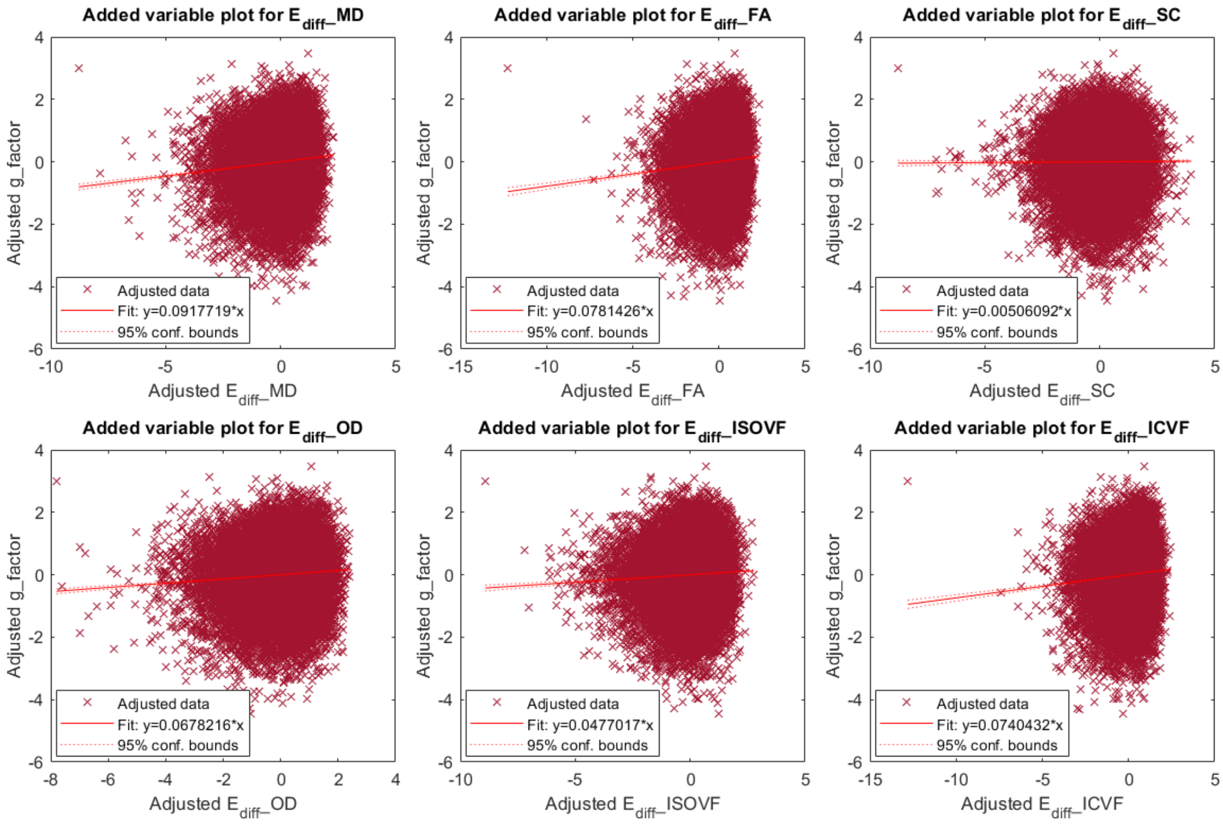

Figure B.8: Scatter plots showing the associations between the diffusion efficiency and the g-factor, adjusted for common covariates, for each of the six network weights.

References

Smith, K. M., Bastin, M. E., Cox, S. R., Valdés Hernández, M. C., Wiseman, S., Escudero, J., and Sudlow, C. (2019). Hierarchical complexity of the adult human structural connectome. *Neuroimage*, 191:205–215.

Smith, K. M. and Smith, J. P. (2024). Statistical complexity of heterogeneous geometric networks. *PLOS Complex Systems*, in press.
